# Systemic thromboembolism including multiple cerebral infarctions with middle cerebral artery occlusion caused by the progression of adenomyosis with benign gynecological tumor: a case report

**DOI:** 10.1186/s12883-021-02045-7

**Published:** 2021-01-11

**Authors:** Ryo Aiura, Sadayoshi Nakayama, Hroo Yamaga, Yu Kato, Hirotake Fujishima

**Affiliations:** 1 Department of Neurosurgery, AOI Universal Hospital, 2-9-1, Tamachi, Kawasaki Ward, 210-0822 Kawasaki City, Kanagawa Japan; 2 Department of Neurosurgery, Showa University Fujigaoka Hospital, 1-30, Fujigaoka, Aoba Ward, 227-8501 Yokohama City, Kanagawa Japan; 3 Department of Neurosurgery, Showa University Northern Yokohama Hospital, 35-1, Chigasaki Chuo, Tsuzuki Ward, 224-8503 Yokohama City, Kanagawa Japan

**Keywords:** Adenomyosis, Benign gynecological tumor, Hypercoagulability, Systemic thromboembolism, Multiple cerebral infarction, Middle cerebral artery occlusion, Endovascular thrombectomy, Hysterectomy

## Abstract

**Background:**

Adenomyosis, a benign gynecological disease, causes cerebral infarction. Similar to Trousseau’s syndrome, it elevates cancer antigen 125 (CA125) and D-dimer levels; causes hypercoagulability; and results in cerebral infarction. However, no case of adenomyosis causing major cerebral artery occlusion and requiring endovascular thrombectomy has yet been reported. We report on a woman with middle cerebral artery occlusion caused by adenomyosis progression with a benign gynecological tumor and recurrent cerebral infarction. She was successfully treated by endovascular thrombectomy and hysterectomy.

**Case presentation:**

A 48-year-old woman with heavy uterine bleeding was transported by ambulance to our hospital. Upon arrival, she presented with impaired consciousness. Laboratory test results revealed decreased hemoglobin (8.2 g/dL) and elevated D-dimer (79.3 µg/mL) levels. Radiological imaging revealed adenomyosis, a left ovarian tumor, multiple uterine myomas, and old and new bilateral renal infarctions. She experienced repeated episodes of excessive menstruation caused by adenomyosis and was scheduled for hysterectomy in 2 months at another hospital. After hospital admission, uterine bleeding stopped. However, 5 days after initial bleeding, she had another episode of heavy uterine bleeding and developed left hemiparesis and dysarthria 20 min later. Brain magnetic resonance imaging revealed bilateral multiple cerebral infarctions indicating right middle cerebral artery occlusion. Thus, endovascular thrombectomy was performed, and anticoagulant therapy was administered. Laboratory test results after thrombectomy revealed elevated CA125 (3536 U/mL) and CA19-9 (892 U/mL) levels. She was at a risk of recurrent heavy uterine bleeding leading to repeated cerebral infarction because of anticoagulant treatment. Therefore, we performed hysterectomy and ovariectomy 11 days after initial bleeding. Histopathological assessment revealed no malignancy. Although she developed asymptomatic pulmonary thromboembolism 14 days after initial bleeding, D-dimer and tumor marker levels returned to normal soon after gynecological surgery. At 15 months post-surgery, she had not experienced further ischemic events.

**Conclusions:**

Adenomyosis with benign gynecological tumors may be associated with elevated D-dimer and tumor marker levels; excessive menstruation; and anemia. It may cause systemic thromboembolism, including cerebral infarction. To our knowledge, no other study has reported that adenomyosis causes major cerebral artery occlusion requiring endovascular thrombectomy. Hysterectomy may be an effective radical treatment of this condition.

## Background

Adenomyosis is not a life-threatening gynecological disease. However, it causes excessive menstruation, dysmenorrhea, and menstrual pain. Adenomyosis has also been reported to elevate cancer antigen 125 (CA125) and D-dimer levels, cause hypercoagulability, and result in development of cerebral infarction (CI), similar to Trousseau’s syndrome that is caused by malignant tumor [1–8]. In previously reported cases, most CIs were caused by peripheral vessel occlusion or stenosis of a major cerebral artery. To the best of our knowledge, no other study has reported that adenomyosis causes major cerebral artery occlusion requiring endovascular thrombectomy. Herein, we present our experience with systemic thromboembolism, including multiple CIs with middle cerebral artery occlusion. It resulted from progression of adenomyosis accompanied by multiple uterine myomas and ovarian endometriosis. Recurrent CI was successfully prevented by endovascular thrombectomy and hysterectomy.

## Case presentation

**Fig. 1:**
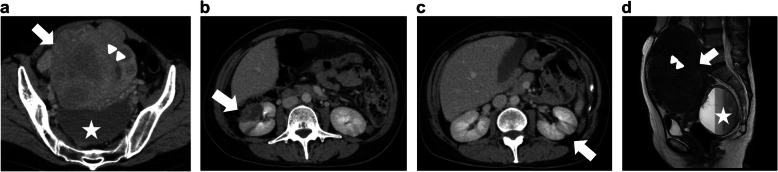
Pelvic imaging on the day of initial bleeding **a**, **d** Pelvic contrast-enhanced computed tomography (CT) and magnetic resonance images show adenomyosis (arrowheads), multiple uterine myomas (arrows), and a left ovarian tumor (stars). **b**-**c** Pelvic contrast-enhanced CT shows old and new bilateral renal infarctions (arrows)

A 48-year-old woman was transported by ambulance to our hospital for heavy uterine bleeding and impaired level of consciousness. She had no underlying conditions except for adenomyosis, for which she was scheduled to undergo hysterectomy in 2 months at another hospital due to recurrent excessive menstruation. Upon arrival, her Glasgow Coma Scale was E4V4M6, and she was pale. She had a fever of 39.1 °C. Laboratory test results revealed a decreased hemoglobin concentration (Hb) (8.2 g/dL; normal 10.8–14.9 g/dL) and elevated D-dimer levels (79.3 µg/mL; normal < 1.0 µg/mL), white blood cell count (WBC) (18,060/µl; normal 3300–8600/µl), and C-reactive protein level (CRP) (20.1 mg/dL; normal < 0.3 mg/dL). Contrast-enhanced computed tomography (CT) and magnetic resonance imaging (MRI) of the pelvic area revealed adenomyosis; left ovarian tumor; multiple uterine myomas; and both old and new bilateral renal infarctions (Fig. 1a-d). She was admitted to the gynecology department and administered an antibiotic agent (200 mg/day cefmetazole sodium) for 5 days. She denied a history of hypertension, hyperlipidemia, or diabetes based on her annual medical checkups as well as a family history of cerebrovascular disease. One day after admission, uterine bleeding stopped, and her consciousness improved. However, her Hb levels furtherly decreased to 6.9 g/dL. Therefore, 4 units of red blood cell concentrates were transfused.

**Fig. 2:**
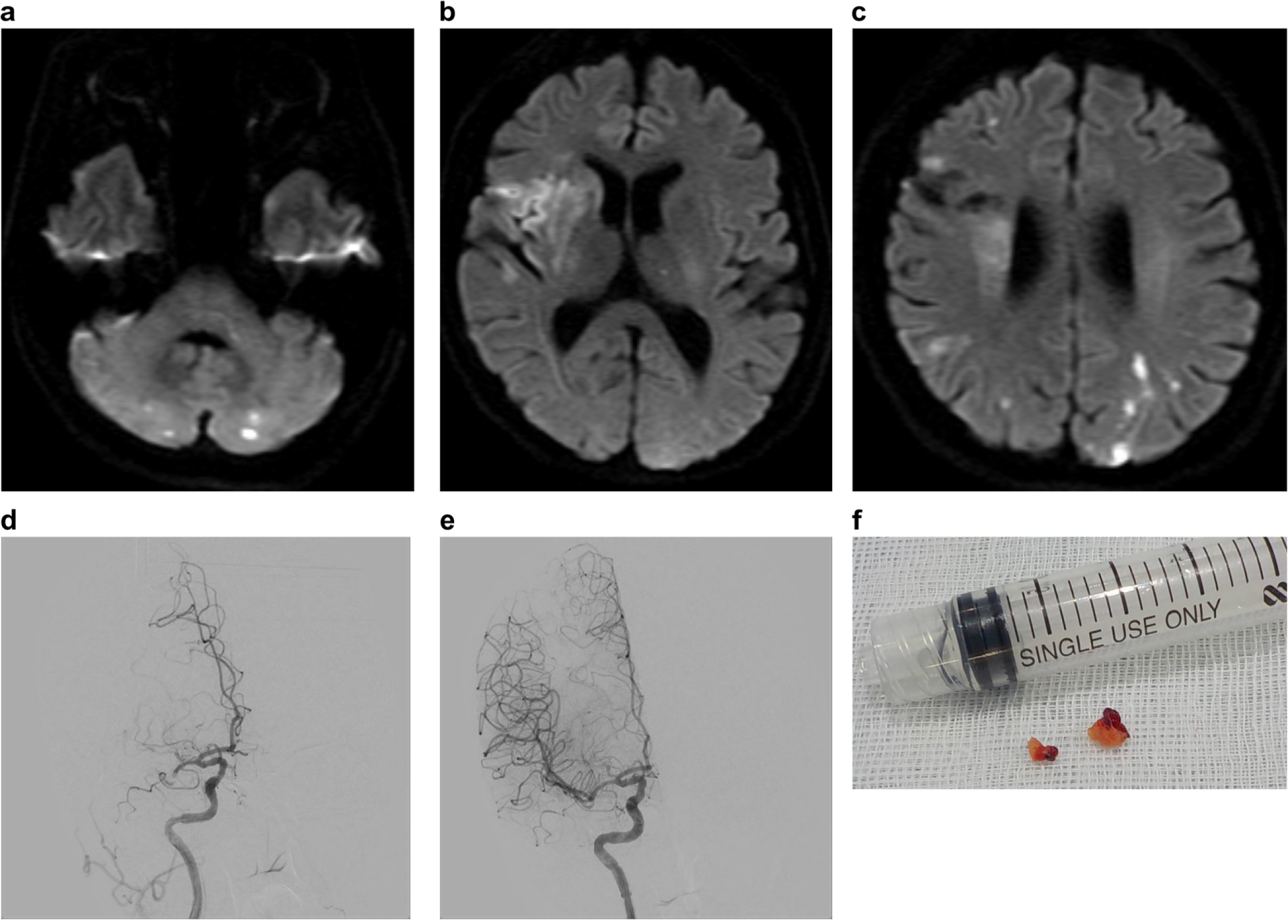
Brain imaging and thrombus collected on the day of the development of cerebral infarctions **a**-**c** Brain magnetic resonance imaging shows bilateral multiple cerebral infarctions indicating right middle cerebral artery occlusion. **d**, **e** Right internal carotid artery angiography shows right cerebral artery occlusion, and thus, we performed endovascular thrombectomy. **f** The collected thrombus

However, 5 days after the initial bleeding, she had another episode of heavy uterine bleeding, and 20 min later, she became unconscious with left hemiparesis and dysarthria. Her National Institutes of Health Stroke Scale (NIHSS) score was 13. Brain MRI revealed bilateral multiple CIs indicating right middle cerebral artery occlusion. In addition, her diffusion-weighted image Alberta Stroke Programme Early CT Score was 6. Endovascular thrombectomy was subsequently performed, and her Thrombolysis in Cerebral Infarction grade was 3 (Fig. 2a-f). After endovascular therapy, her NIHSS score improved to 2. Heparin was administered as anticoagulant therapy to prevent CI. Transesophageal echocardiography did not reveal any evidence of patent foramen ovale or valvular vegetation. Moreover, a 24-h Holter electrocardiogram did not show atrial fibrillation or any other arrhythmia. Due to her underlying gynecological tumor, additional laboratory testing for tumor markers were performed the day after thrombectomy. These results revealed elevated CA125 (3536 U/mL; normal < 35.0 U/mL) and CA19-9 (892 U/mL; normal < 37.0 U/mL) levels. Levels of protein S, protein C, lupus anticoagulant, Factor V coagulation activity, anticardiolipin antibody, anti-cardiolipin-beta-2-glycoprotein complex antibody, and carcinoembryonic antigen were within normal range.

**Fig. 3:**
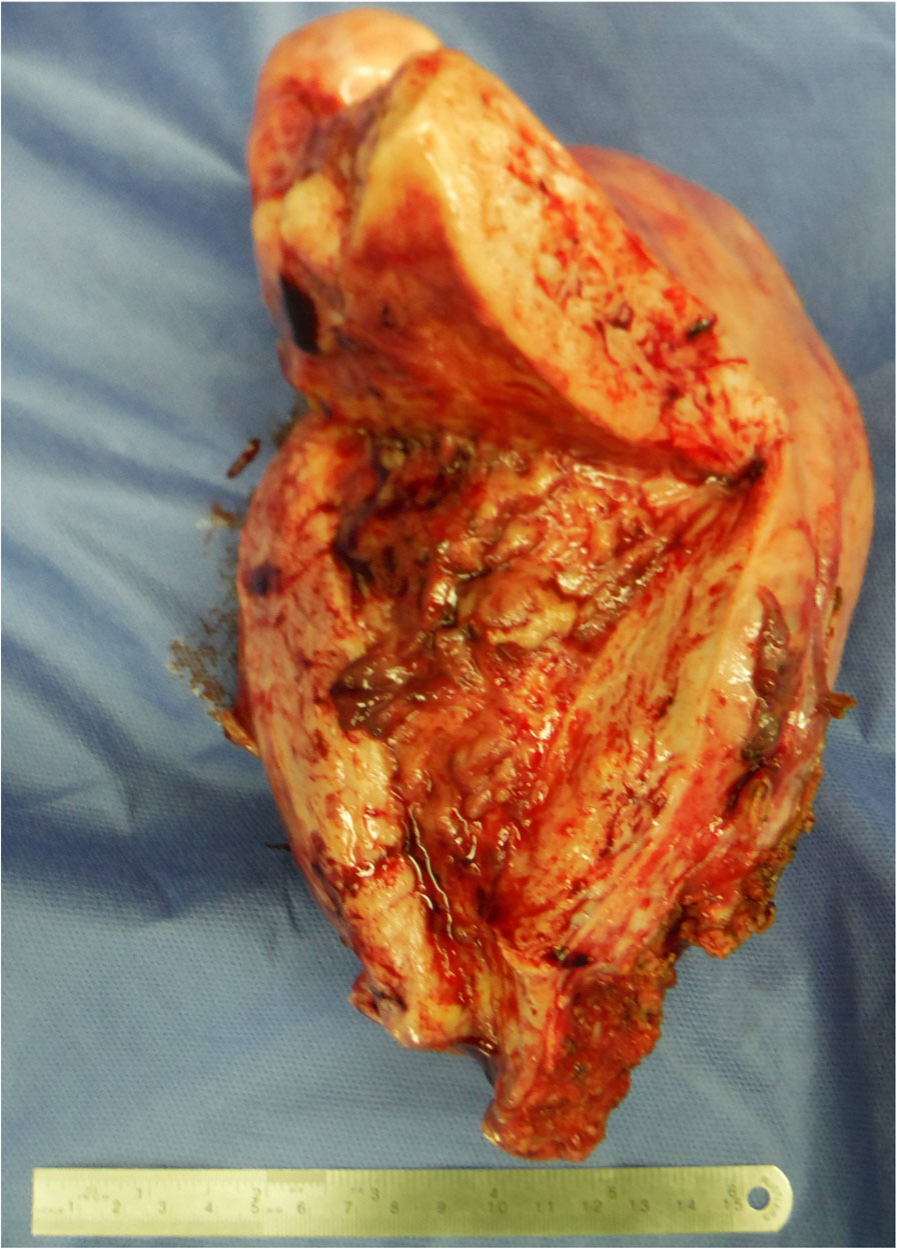
The resected uterus

**Fig. 4:**
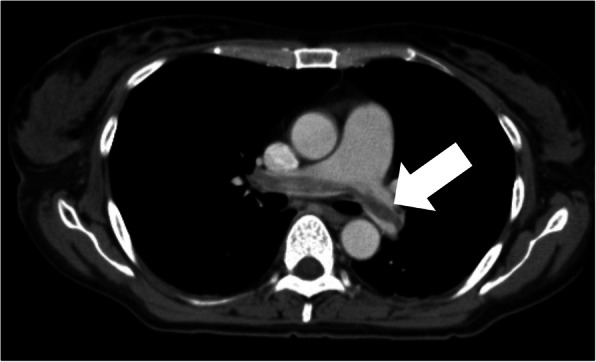
Chest contrast-enhanced computed tomography shows a giant thrombus in the pulmonary artery (arrow)

**Fig. 5:**
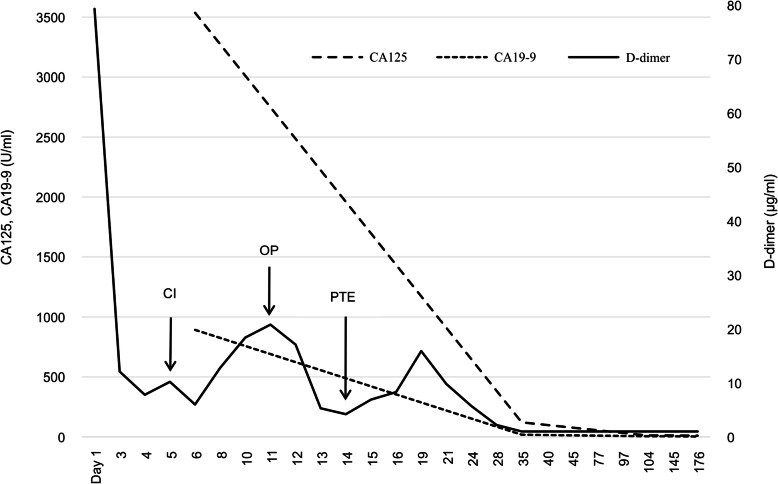
Changes in D-dimer, CA125, and CA19-9 levels on the day after the initial bleeding. Abbreviations: CI, cerebral infarction; OP, operation day; PTE, pulmonary thromboembolism.

**Fig. 6:**
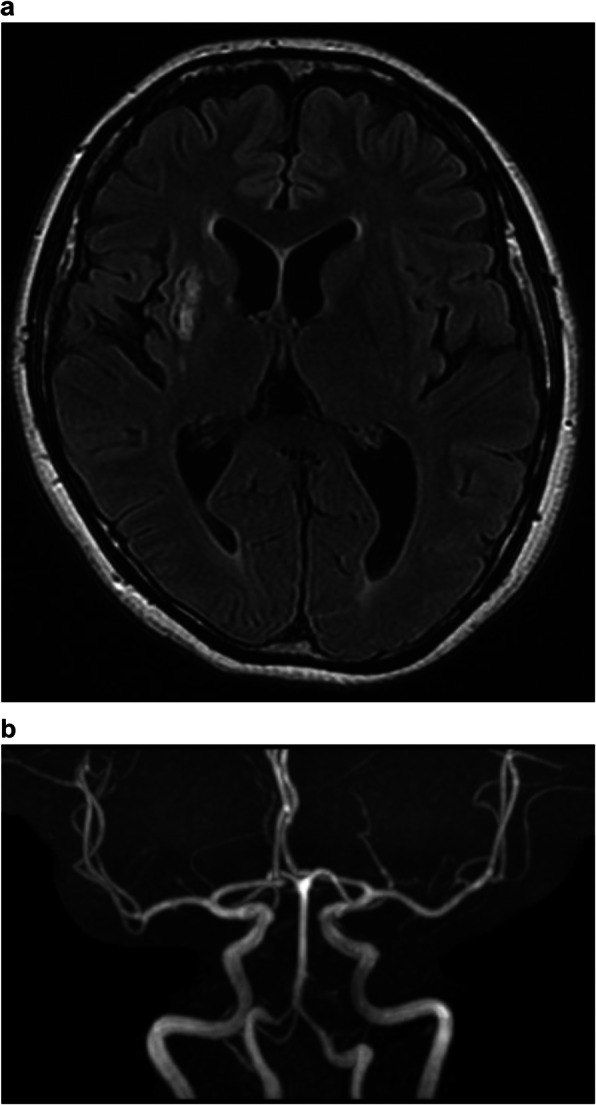
Brain magnetic resonance image at 11 months postoperatively (**a**-**b**) No cerebral infarction can be observed, and the major cerebral arteries are well visualized

We were concerned that using heparin might cause heavy uterine bleeding and that could have led to repeated CI. Since she was scheduled for a hysterectomy in 2 months at another hospital, with her consent, hysterectomy and ovariectomy were performed earlier, 11 days after the initial bleeding (Fig. 3). We discontinued heparin therapy after surgery. Histopathological assessment revealed no malignancy, and diagnoses of adenomyosis, uterine myoma, and ovarian endometriosis were established. On day 14 after the initial bleeding, whole-body contrast-enhanced CT revealed pulmonary thromboembolism, (Fig. 4) which was asymptomatic, and heparin was re-administered. On day 20 after the initial bleeding, because her pulmonary thromboembolism tended to dissolve, we switched the anticoagulant therapy from heparin to edoxaban 60 mg/day for 6 months. The patient’s D-dimer levels dropped immediately after surgery, and CA125 and CA19-9 levels returned to normal range (12.0 U/mL and 5.6 U/mL, respectively) 3 months after surgery (Fig. 5). Since her symptoms had improved and her NIHSS score was 0, she was discharged with a modified Rankin Scale score of one, 42 days after she experienced CI. The patient had not experienced additional ischemic events 15 months postoperatively (Fig. 6a, b).

## Discussion and conclusions

Herein, we report the case of a patient with adenomyosis accompanied by multiple uterine myomas and ovarian endometriosis that led to systemic thromboembolism, including multiple CIs with right middle cerebral artery occlusion. We suggested that her untreated adenomyosis accompanied by uterine myoma and ovarian endometriosis was associated with elevated D-dimer, CA125, and CA19-9 levels and caused thromboembolic CI, similar to Trousseau’s syndrome.

A systematic literature search was conducted to identify cases of CI associated with adenomyosis through a PubMed search between November 2012 and September 2020 with the keywords “adenomyosis” and “cerebral infarction,” and 15 cases including our case were identified (Table 1) [1–8]. Table 1 demonstrates that most patients had decreased Hb and elevated D-dimer and CA125 levels, which occurred during menstruation in half of the patients. Four of the 15 patients had major cerebral artery occlusion. Five of the 15 patients eventually underwent hysterectomy to prevent recurrent CI, and there was no subsequent recurrence of CI in these patients. Patients having fever were reported in 3 cases [2, 5, 7]. Furthermore, systemic embolism involving the fingers, kidneys, and spleen, as well as thrombi in the brachiocephalic trunk and left subclavian artery were reported in 3 cases [1, 2]. In this patient (Table 1; case 15), CA125 and D-dimer levels were significantly more elevated than in any other case. In addition, systemic embolism, including pulmonary thromboembolism, bilateral renal infarctions, and multiple CIs, occurred. Out of the 14 patients in the literature, there was no case in which adenomyosis was accompanied by a benign gynecological tumor such as uterine myoma or ovarian endometriosis or in which endovascular thrombectomy was required.

**Table 1:** Clinical profiles of previous cases of cerebral infarction associated with adenomyosis

Case	Age	Occurrence during menstruation	CA125 (U/mL)	CA19-9 (U/mL)	D-dimer (µg/mL)	Hb (g/dL)	Major cerebral artery	Treatment for CI	Treatment for denomyosis	Recurrence of CI
1 [1]	45	-	159	-	1.1	8.4	Normal	Heparin, antiplatelet therapy	GnRH agonist	-
2 [1]	44	-	-	-	-	7	Normal	Heparin, warfarin	GnRH agonist	-
3 [1]	50	+	42.6	-	0.57	6.9	Normal	Aspirin	GnRH agonist	-
4 [1]	42	+	1750	-	6	8.6	Normal	Antiplatelet therapy	GnRH agonist	+
								Heparin, warfarin	GnRH agonist	-
5 [6]	59	-	334.8	-	7	-	-	Antithrombotic therapy	-	-
6 [7]	48	-	901	1791	1.9	8.5	Occlusion of the left M1 of MCA	Heparin, warfarin	Hysterectomy	-
7 [8]	49	-	379	69.2	3.99	9.9	Normal	Anticoagulant	Hysterectomy	-
8 [2]	44	+	2115	1824	17	10.3	Severe stenosis in the right M2of MCA	Heparin, rivaroxaban	GnRH agonist	+
									Hysterectomy	-
9 [3]	42	-	395	-	1.4	-	Occlusion of the left M2 of MCA	Warfarin	-	-
10 [3]	50	-	143	-	3.7	-	After occlusion of the left M1 of MCA, partial reperfusion of MCA	Rivaroxaban	-	-
11 [4]	34	+	937.1	462.5	1.05	13.4	Normal	-	-	-
12 [4]	37	+	735.7	43.2	12.04	10.8	Normal	-	-	-
13 [4]	46	+	546.5	1076.6	2.34	12.1	Stenosis of the right PCA	-	Hysterectomy	-
14 [5]	34	+	937.7	-	27.4	11.2	Severe stenosis in the right M1 of MCA	Heparin, antiplatelet therapy	-	-
15	48	+	3536.2	892.1	79.3	8.2	Occlusion of the right M1 of MCA	Heparin, edoxaban Endovascular thrombectomy	Hysterectomy	-

Abbreviations: *CA* cancer antigen; *CI* cerebral infarction; *GnRH* gonadotropin-releasing hormone; *Hb* hemoglobin concentration; *MCA* middle cerebral artery; *PCA* posterior cerebral artery

The levels of tumor markers, such as CA125, that fluctuate during the menstrual cycle reach a peak during menstruation [9]. Severe adenomyosis with a uterine size > 12 weeks’ gestation or volume beyond 240 cm^3^ has been shown to raise CA125 levels [10]. CA125 levels have been observed to be significantly elevated in patients with endometriosis accompanied by adenomyosis, and CA19-9 levels increased significantly following the progression of endometriosis [11]. Furthermore, patients with both uterine myoma and ovarian endometriosis, which were at a risk of developing cancer, have been shown to have elevated CA125 and CA19-9 levels [12]. Therefore, we considered that CA125 and CA19-9 levels may easily have increased because of menstruation and adenomyosis accompanied by uterine myoma and ovarian endometriosis.

Additionally, elevated CA125 levels may be associated with CI [13] and they correlate with D-dimer levels under a high tumor burden [14]. Carcinoma mucins, such as CA125 and CA19-9 promote thrombosis through increased leukocytes and platelets activities [15, 16]. Thus, carcinoma mucins were thought to play an important role in hypercoagulation. Yin et al. [4] reported that patients with adenomyosis had a higher risk of developing CI during menstruation. Aso et al. [2] also suggested that middle-aged women with adenomyosis were at a high risk of CI when D-dimer, CA125, and CA19-9 levels were elevated. Moreover, they concluded that anticoagulant therapy, including direct oral anticoagulants, could not prevent CI. However, hysterectomy was the most effective prevention of CI.

Furthermore, Zhao et al. [5] reported that infection was a potential risk for developing acute CI with adenomyosis. Since our patient also had fever and her laboratory tests demonstrated elevated WBC and CRP, infection might have furthered her hypercoagulability in addition to both menstruation and elevated CA125, CA19-9, and D-dimer levels, resulting in CI with major cerebral artery occlusion.

In the present case, we suggest that the patient’s tumor markers, such as CA125 and CA19-9, were significantly elevated due to menstruation and severe adenomyosis accompanied by multiple uterine myomas and ovarian endometriosis that were shown to be benign on histopathological examination [9–12]. We also suggest that these carcinoma mucins activated coagulation, caused thromboembolism, and resulted in the development of CI and systemic embolism [13–16]. In the case of CI due to thromboembolism, we would expect that endovascular thrombectomy would be effective. However, mechanisms for thromboembolism caused by a benign gynecological disease that elevated CA125, CA19-9, and D-dimer levels are not fully elucidated. Thus, further studies are required.

In conclusion, this case suggests that adenomyosis accompanied by a benign gynecological tumor and menstruation is associated with elevated D-dimer, CA125, and CA19-9 levels and increases the risk of systemic embolism including CI. Moreover, this clinical situation may cause major cerebral artery occlusion requiring endovascular thrombectomy, and hysterectomy may be the most effective therapy to prevent CI.

## Data Availability

Not applicable.

## Abbreviations

CA: Cancer antigen
CI: Cerebral infarction
CRP: C-reactive protein level
CT: Computed tomography
Hb: Hemoglobin concentration
MRI: Magnetic resonance imaging
NIHSS: National Institutes of Health Stroke Scale
WBC: White blood cell count
